# Future Orientation Among Children Affected by Parental HIV in China: An Exploratory Analysis of Complex Interactions

**DOI:** 10.3389/fsoc.2022.899537

**Published:** 2022-07-07

**Authors:** Heather L. McDaniel, Sayward E. Harrison, Amanda J. Fairchild, Xiaoming Li

**Affiliations:** ^1^School of Education and Human Development, University of Virginia, Charlottesville, VA, United States; ^2^Department of Psychology, College of Arts and Sciences, University of South Carolina, Columbia, SC, United States; ^3^South Carolina SmartState Center for Healthcare Quality, Arnold School of Public Health, University of South Carolina, Columbia, SC, United States; ^4^Department of Health Promotion, Education, and Behavior, Arnold School of Public Health, University of South Carolina, Columbia, SC, United States

**Keywords:** resilience, future orientation, children affected by HIV, social support, stigma

## Abstract

We utilized an exploratory analytic approach to examine predictors of children's future beliefs, an internal asset associated with resilience among children affected by HIV, with emphasis on complex interactions among multisystem factors. Children (*N* = 1221) affected by parental HIV in China reported on psychosocial functioning, as well as internal, familial, and community resilience assets. Exploratory data analysis was conducted using a binary segmentation program. Six binary splits on predictors accounted for 22.78% of the variance in future expectation, suggesting interactions between children's perceived control of their future, loneliness, caregiver trust, and social support. Four binary splits accounted for 23.15% of the variance in future orientation, suggesting multiway interactions between control of the future, loneliness, social support, and perceived stigma. Findings suggest combinations of resilience factors are associated with children's positive future beliefs. Implications for screening, prevention, and intervention among Chinese children affected by parental HIV are discussed.

## Introduction

Since the beginning of the human immunodeficiency virus (HIV) epidemic in the 1980s, ~76 million individuals have acquired HIV, and ~38 million individuals are currently living with the virus [Joint United Nations Programme on HIV/AIDS (UNAIDS, 2020)]. In the past two decades, the number of children infected with HIV through mother-to-child transmission has been dramatically reduced through expanded HIV testing among pregnant women and increased access to antiretroviral therapy for pregnant and breastfeeding women living with HIV (Joint United Nations Programme on HIV/AIDS, 2011; Panel on Treatment of Pregnant Women with HIV Infection Prevention of Perinatal Transmission, 2020). Currently, ~1.8 million children (i.e., <15 years of age) are estimated to be living with HIV worldwide (Joint United Nations Programme on HIV/AIDS, 2020). However, many more children are indirectly impacted through parental HIV infection or parental death from Acquired Immune Deficiency Syndrome (AIDS)—particularly in low- and middle-income nations (WHO, 2013).

In China, the earliest documented HIV cases occurred in the 1980s among people with histories of injection drug use and hemophiliacs; these were followed by a rapid rise in HIV cases in central China in the mid-1990s caused primarily by unhygienic commercial blood and plasma collection practices (Wu et al., 1995, 2019; Neild and Gazzard, 1997). Multiple rural farming communities across central China experienced devastating largescale HIV outbreaks over a relatively short period of time (Wu et al., 2001; Sun et al., 2010). By 1995, the outbreaks were identified, resulting in the closure of blood and plasma collection centers in the region and the establishment of nationwide blood collection facilities (Sun et al., 2010). However, by this time, HIV had spread widely within many rural communities in central China, with HIV prevalence rate estimates ranging from 10 to 60% among plasma donors across local villages (Wu et al., 2001; China Ministry of Health UN Theme Group on HIV/AIDS in China, 2003; Li et al., 2010). In addition, the majority of paid plasma donors were young and middle-aged adults between the ages of 20 and 50 years (Wu et al., 2001); thus, many individuals who acquired HIV through plasma donation had spouses and children—creating unique psychosocial challenges for affected families.

Parental illness and parental death have profound impacts on children's development and are heightened within the context of HIV—a highly stigmatized health condition (Cluver et al., 2013). Children who are made vulnerable through parental HIV face a range of challenges, including the potential for orphanhood, if parents are unable to access and adhere to antiretroviral therapy, which did not become widely available in China until the 2000s with the advent of the Chinese government's “Four Frees and One Care” policy (Sun et al., 2010). This policy was designed to provide free access to HIV medications, free voluntary counseling and HIV testing, free prevention of mother-to-child transmission, free schooling for children affected by parental HIV, and social relief for affected families (Sun et al., 2010). Aside from the threat of orphanhood, children made vulnerable by parental HIV are often separated from their HIV-positive parents (i.e., due to parental illness, due to stigma and shame within extended families), and economic insecurity within HIV-affected families is common (Foster and Williamson, 2000; Li et al., 2008; Sherr et al., 2008).

Much of the seminal work exploring the impact of parental HIV on children's mental health has been conducted in central China in partnership with impacted rural communities—notably in Henan province. Chinese children affected by parental HIV have been shown to be at-risk for internalizing disorders (e.g., anxiety, depression), adjustment problems and school-related challenges (Fang et al., 2009; Guo et al., 2012; Du et al., 2015). A systematic review of the impact of parental HIV on children's psychosocial wellbeing found inconsistent effects of children's gender and age on psychosocial outcomes; some studies have found that boys are more susceptible to negative mental health outcomes (e.g., feelings of hopelessness, poor quality of life) than girls following the AIDS-related death of a parent, while other studies have shown no gender differences (Chi and Li, 2013).

Notably, three clusters of risk factors have been consistently identified across contexts to heighten risk for children affected by parental HIV: exposure to traumatic or stressful life events, experience of HIV-related stigma, and socioeconomic disadvantage (Chi and Li, 2013). Within the context of the central China HIV outbreak of the 1990s, children affected by parental HIV are at high risk for trauma exposure (Li et al., 2009). When compared to children not affected by HIV, children with parents who were living with HIV or who had died from AIDS were more likely to have experienced a range of trauma—not only parental death or illness, but also serious accidents and injuries, parental separation or divorce, removal from their biological family, child maltreatment, child sexual abuse, assault, and property crimes (Li et al., 2009).

Children affected by parental HIV also frequently experience high levels of stigma. HIV-related stigma has been defined as the prejudice, discounting, discrediting, and discrimination directed toward individuals living with HIV and groups associated with the virus (Herek, 1999). Importantly, stigma is not only an individual experience but also a socio-cultural process that creates and maintains inequity in cultural standing and social class and contributes to longstanding inequities in systems and structures of power (Foucault, 1977, 1990; Parker and Aggleton, 2002). Children whose parents acquired HIV during the Henan outbreak have reported experiencing significant public stigma from their communities. Both perceptions of community-held stigma and personal experiences of stigma are associated with poorer psychosocial functioning (Lin et al., 2010).

Fortunately, not all children impacted by parental HIV demonstrate adverse outcomes over time, and efforts to identify and cultivate resilience factors among children affected by HIV have gained momentum in the past decade (Chi et al., 2014a). Here we define resilience as, “the process and outcome of successfully adapting to difficult or challenging life experiences, especially through mental, emotional, and behavioral flexibility and adjustment to external and internal demands” (VandenBos, 2007). Notably, Li et al. (2015) have developed a conceptual framework that delineates resilience factors across three levels—child, family, and community—that can help buffer the negative impacts of parental HIV for children. Specifically, the model integrates concepts from Bronfenbrenner's (1979) ecological systems theory of human development and from theories of resilience (Luthar et al., 2000; Masten, 2011; Ungar et al., 2013) to highlight protective factors across child (e.g., positive beliefs, coping skills), family (e.g., secure caregiver attachment, positive parenting skills), and community domains (e.g., peer and community support) that can reduce risk and promote positive developmental outcomes for children made vulnerable by HIV. This framework of psychological resilience for children affected by HIV (Li et al., 2015) builds on existing calls for moving away from deficit-based perspectives in order to adopt resilience or strengths-based approaches to mitigate the negative impacts of parental HIV on children's development (Skovdal and Daniel, 2012; Betancourt et al., 2013; Li et al., 2015).

In terms of individual resilience assets, children's beliefs—particularly beliefs about the future—have been identified as salient to a range of cognitive and behavioral processes that impact developmental outcomes. Children who believe they will be successful in the future are more likely to persist when confronted with challenges (Bandura, 1977a,b; Masten, 2011), and positive beliefs about the future are a key protective factor associated with reduced engagement in risky behaviors for children and adolescents (Robbins and Bryan, 2004; Herrenkohl et al., 2005; Peters et al., 2005; Cabrera et al., 2009). At-risk children who have consistently high future orientation or who show positive growth in future orientation during adolescence have been shown to have greater achievement of key developmental milestones in adulthood (i.e., greater income, social capital, employment; Oshri et al., 2018). In addition, positive future orientation mitigates the impact of adversity on child outcomes (Cui et al., 2020).

Because children affected by parental HIV often lack family support and community resources to set them up for future success, their own beliefs and motivations about the future are very important for maintaining mental health and wellbeing as they cope with the adversity associated with familial HIV (Auslander et al., 1998; Li et al., 2015). In fact, future orientation has also been found to be an important mediator of the relationship between trauma exposure and psychosocial functioning among children affected by HIV in rural central China (Zhang et al., 2009). Thus, within the uniquely stigmatizing context of HIV, children's beliefs about the future and related constructs, such as sense of control over their environment, self-efficacy, and self-esteem, are potentially vital resilience assets (Wang et al., 2012; Li et al., 2015). And given a more recent shift from a deficit focus (e.g., on psychopathology outcomes) to a more resilience-based focus (i.e., to more positive outcomes), it will be essential to understand how these individual assets might be promoted. To complement research on how these internal assets are protective against poor mental health outcomes, it will also be critical to study these resilience factors as outcomes, to better understand which youth might be at risk for low levels of these internal assets, as well as how these essential assets might be bolstered.

Beyond individual resilience assets, social support is critical for children's development. For example, previous research among Chinese children affected by parental HIV indicates that, when controlling for key covariates (e.g., gender, age, socioeconomic status [SES], orphanhood status), having a trusting relationship with a caregiver is significantly associated with a range of positive psychosocial outcomes such as self-esteem, school interest, social skills, and hopefulness about the future; Zhao et al., 2011). In addition, perceived social support from friends, family, and teachers has been identified as a significant predictor of positive psychosocial adjustment among children affected by parental HIV in Henan, China, independent of children's gender, age, SES, and orphanhood status (Hong et al., 2010).

While multi-level resilience factors have been identified as protective for psychosocial development among Chinese children made vulnerable by HIV, greater understanding is needed of how risk and resilience factors interact to promote the internal assets of youth. Here we focus on the outcome future orientation, which has been linked to other positive developmental outcomes and is likely an essential asset to target in preventive interventions for this population (Bandura, 1977b; Masten, 2011; Oshri et al., 2018). It has long been recognized that interactions amongst risk and protective factors are integral to models of resilience for at-risk children (Masten, 2001). For children affected by parental HIV, internal assets, family resources, and community resources interact to buffer or mitigate the deleterious effects associated with parental HIV (e.g., stigma, trauma; Li et al., 2015). The current research uses an exploratory analytic approach to examine salient protective factors from Li et al. (2015) resilience framework to predict future orientation outcomes. Specifically, we leverage the SEARCH algorithm, a binary segmentation program for exploratory data analysis, that aims to account for unexplained variance in a defined outcome (Morgan and Sonquist, 1963; Sonquist et al., 1973; Morgan, 2005). SEARCH has been coined an “automatic interaction detector”, given utility in detecting complex, multiway interactions (Sonquist et al., 1973). Using SEARCH, we aim to improve prediction of an important resilience-related asset, positive future orientation, by splitting all possible candidate predictors in a sample of Chinese children impacted by parental HIV. By using candidate predictors informed by the resilience framework outlined by Li et al. (2015), the current research places an emphasis on understanding the manner in which multisystem factors interact to bolster this important internal asset among children affected by parental HIV.

## Materials and Methods

### Sample and Procedures

Data for the current study are derived from the baseline data collection of a broader project to understand psychosocial adjustment of children affected by parental HIV in China. Participants include children and adolescents from Henan, China, a province that has been highly impacted by the HIV epidemic due to unhygienic blood and plasma donation practices in the 1990s (Wu et al., 1995, 2001; Neild and Gazzard, 1997). Children aged 6 to 18 years were eligible to participate in the current study. Additionally, children had to either have a biological parent living with HIV or be an AIDS orphan (i.e., lost one or both parents to an AIDS-related death) to be eligible for this secondary data analysis.

Children were recruited for the study in partnership with local government-funded orphanages and group homes, as well as local village leaders. A total of four government-funded orphanages and eight village-level group homes took part in recruitment efforts. In addition, the research team worked with village leaders to generate lists of families affected by the HIV outbreak (i.e., in which one or both parents had acquired HIV) and to identify lists of families caring for local AIDS orphans. Working in close partnership with community leaders and stakeholders, the research team approached orphanages, homes, and families about the study opportunity, and children were invited to take part, with children providing assent and parents or government-appointed guardians providing consent. The study protocol, including consenting process, was approved by institutional review boards at Wayne State University in the United States and Beijing Normal University in China.

This recruitment process yielded a sample for the current study that consisted of 1,221 Chinese children impacted by parental HIV. Child demographic information is shown in Table 1. Each child participating in the study completed a paper-pencil survey battery in Chinese. The battery included demographic items as well as scales assessing psychosocial factors. The entire battery took around 75 to 90 minutes to complete, depending on the age of the child. Research staff read items aloud to younger children and those with reading difficulties, and breaks were provided to children as needed. Research staff also provided clarification if children had questions about items. All children who participated received a small gift at the completion of the survey battery in appreciation of their time.

**Table 1:** Child demographic information.

	**Frequency**	**Percentage**
Male	622	50.9
Children in orphanage care	176	14.4
Children in kinship care	579	47.4
Children living with an affected parent	466	38.2
Age		
6	1	0.1
7	5	0.4
8	26	2.1
9	65	5.3
10	114	9.3
11	122	10.0
12	188	15.4
13	176	14.4
14	194	15.9
15	158	12.9
16	131	10.7
17	28	2.3
18	3	0.2
Missing	10	0.8

### Measures

Selection and translation (when needed) of scales used in the current study have been described in detail elsewhere (Fang et al., 2009). Briefly, the research team initially identified scales that were available in Chinese and had already been validated with Chinese samples. Chinese scales were not initially available for some variables of interest. In these instances, a Chinese-English bilingual research team used a forward-backward translation process to translate English scales into Chinese; items and wording were reviewed by a group of Chinese faculty in psychology and education departments at the partnering Chinese university to ensure cultural and developmental appropriateness. All translated scales were piloted with Chinese children to check for comprehension and ensure that meaning remained intact.

#### Outcome Measures

The primary outcomes of the current study were two measures assessing children's beliefs about their future.

##### Future Expectation

Children's future expectations were assessed with a modified Chinese scale based on the *Children's Future Expectation Scale* (Bryan et al., 2005). This modified scale consisted of six items rated on a five-point Likert scale that assessed children's certainty of accomplishing generalized outcomes in the future. Example translated items include, “How sure are you that you can handle the problems that might come up in your life in the future?”, “How sure are you that you will have interesting things to do in your life?”, and “How sure are you that you will have a happy life?” Higher mean scores indicated greater certainty in generalized outcomes in the future. The measure demonstrated good internal consistency in the analytic sample (α = 0.84).

##### Future Orientation

Children's future orientation was measured with a translated scale consisting of four items rated on a four-point Likert scale (Whitaker et al., 2000). The four items captured aspects of specific future socioeconomic attainment, for example, “How likely do you think it is that you will graduate from high school or get your GED some day?” and “How likely do you think it is that you will get a good job some day?”. Higher mean scores indicated a more positive future orientation specifically related to socioeconomic attainment. Cronbach's alpha showed adequate internal consistency (α = 0.78) for the future orientation scale.

#### Predictor Variables

Several predictor variables were selected for potential inclusion in the binary segmentation model that corresponded to the difficulties associated with parental HIV, as well as the child, family, and community resources that are theorized to be assets that promote resilience among these vulnerable children (Li et al., 2015). Descriptive statistics for all potential predictors are presented in Table 2.

**Table 2:** Descriptive statistics and correlations for potential SEARCH predictors and outcomes.

**Scale**	**Mean (SD)**	**Percent Missing**	**1**	**2**	**3**	**4**	**5**	**6**	**7**	**8**	**9**	**10**	**11**	**12**	**13**	**14**
1. Future expectation	3.02 (0.92)	0.98	1.00													
2. Future orientation	2.80 (0.74)	0.90	0.47^**^	1.00												
3. Perceived control of the iuture	2.93 (0.50)	1.15	0.43^**^	0.50^**^	1.00											
4. Life incidence of traumatic events	2.93 (2.53)	3.03	−0.01	−0.05	−0.08^**^	1.00										
5. Stigma against children affected by AIDS scale	2.13 (0.70)	0.57	−0.16^**^	−0.14^**^	−0.18^**^	0.12^**^	1.00									
6. Experienced stigma scale	1.61 (0.60)	3.44	−0.10^**^	−0.10^**^	−0.25^**^	0.28^**^	0.31^**^	1.00								
7. Depression scale for children	0.97 (0.43)	0.16	−0.10^**^	−0.09^**^	−0.22^**^	0.30^**^	0.23^**^	0.53^**^	1.00							
8. Trusting relationship questionnaire	2.51 (0.71)	0.16	0.28^**^	0.24^**^	0.27^**^	0.06^*^	−0.11^**^	0.05	0.01	1.00						
9. Children's loneliness scale	2.52 (0.70)	0.33	−0.39^**^	−0.31^**^	−0.43^**^	0.05	0.20^**^	0.32^**^	0.31^**^	−0.25^**^	1.00					
10. Perceived social support–family	3.26 (1.04)	0.49	0.27^**^	0.21^**^	0.21^**^	−0.02	−0.14^**^	−0.06^*^	−0.09^**^	0.40^**^	−0.23^**^	1.00				
11. Perceived social support–friend	3.00 (1.01)	0.66	0.24^**^	0.22^**^	0.18^**^	0.00	−0.09^**^	−0.04	−0.03	0.37^**^	−0.23^**^	0.59^**^	1.00			
12. Perceived social support–teacher	2.93 (1.07)	0.74	0.20^**^	0.17^**^	0.10^**^	−0.04	−0.10^**^	0.02	−0.03	0.26^**^	−0.13^**^	0.52^**^	0.51^**^	1.00		
13. Perceived social support–other	3.16 (1.06)	0.49	0.25^**^	0.23^**^	0.23^**^	−0.01	−0.15^**^	−0.08^**^	−0.05	0.37^**^	−0.26^**^	0.63^**^	0.63^**^	0.47^**^	1.00	
14. Schoolagers coping strategies inventory	1.73 (0.40)	0.66	0.05	0.01	−0.05	0.30^**^	0.11^**^	0.34^**^	0.32^**^	0.28^**^	0.10^**^	0.12^**^	0.19^**^	0.10^**^	0.12^**^	1.00

* *Correlation is significant at the 0.05 level*.

** *Correlation is significant at the 0.01 level*.

##### Demographic Covariates

Children reported on a variety of demographic characteristics including gender, age, and county of residence; these were all specified as potential predictors for the model.

##### Impact of Parental HIV

The following covariates were included as potential predictors to reflect some of the ways in which a child may be impacted that may reduce the likelihood of positive resilience related outcomes.

###### Orphan Status.

The sample included children made vulnerable by parental HIV (i.e., one or both parents are HIV-positive), single orphans (i.e., one parent has died due to AIDS), and double orphans (i.e., both parents have died due to AIDS). The potential additive effect of being an orphan, in comparison to a vulnerable child living with an HIV-positive parent, was specified as a potential predictor.

###### Living Situation.

Previous literature has suggested that AIDS orphans have different psychosocial outcomes based on their living situation (i.e., institutional care vs. familial care; Fang et al., 2009). Therefore, this variable was included as a potential predictor of resilience related outcomes.

###### Life Incidence of Traumatic Events (LITE).

The LITE (Greenwald and Rubin, 1999) was utilized in the current project to assess children's exposure to traumatic events. The original 17-item LITE was utilized in a translated format with the addition of one item specifically associated with being impacted by parental HIV. Example items include being “taken away from family” and “been hit, whipped, beaten, or hurt by someone”. The focal composite score from this measure was the total number of types of incidences that the child endorsed experiencing. Higher scores indicated that the child endorsed experiencing more types of traumatic events.

###### The Stigma Against Children Affected by AIDS Scale.

This scale (Zhao et al., 2010) consisted of 10 Likert-style items aimed at assessing the child's perspective of public stigma toward children impacted by parental HIV. Items were rated on a four-point scale, with higher scores suggesting that the child perceived greater public stigma against children affected by HIV. Example items include, “Most people think AIDS orphans should leave their villages” and “Most people do not think AIDS orphans deserve sympathy”. A mean score was used as a predictor in the current project, with higher scores indicating greater perceived stigma against children impacted by parental HIV. This scale demonstrated good internal consistency (α = 0.87).

###### Experienced Stigma Scale.

This scale (Zhao et al., 2012) consisted of 14 Likert-style items that assessed how frequently children had experienced a range of stigmatizing acts related to being affected by parental HIV. Items were rated on a five-point scale ranging from “never” to “always”. Example items include, being “physically abused by other people or other kids” and “kids stopped playing with me”. In the current study, a mean score was employed as a predictor, with higher scores indicating increased experiences of stigma. This scale demonstrated good internal consistency (α = 0.88).

###### Center for Epidemiological Studies Depression Scale for Children.

This measure (Fendrich et al., 1990) was a 20-item, self-report of symptoms of depression during the past week. Children responded on a four-point scale. Example items include, “I felt down and unhappy” and “I felt like crying”. A mean score was used in the current study, with higher scores indicating greater depressive symptoms. This measure demonstrated good internal consistency (α = 0.81).

###### Children's Loneliness Scale.

The Chinese-adapted version of this scale (Asher et al., 1984; Wang, 1993) was utilized which consisted of 24 total items. Of the 24 items, eight items were unrelated items and not utilized to calculate a score (e.g., “I play sports a lot”). Sixteen items were child self-reported items assessing a child's perceived level of loneliness and social dissatisfaction. Items were rated on a five-point scale ranging from “strongly disagree” to “strongly agree”. Example items include, “I have nobody to talk to” and “I have lots of friends”, which was reverse scored. A mean score of the 16 items was used in the current study, with higher scores reflecting increased levels of loneliness and social dissatisfaction. Alternatively, lower scores demonstrated social strengths (e.g., “I'm good at working with other children” [reverse scored]). This measure demonstrated good internal consistency (i.e., α = 0.80).

##### Resilience Assets—Children's Internal Resources

The internal, possible resilience-promoting assets were assessed by the following scales:

###### Schoolagers Coping Strategies Inventory.

This measure (Ryan-Wenger, 1990) was a 26-item scale assessing the child's frequency of utilization of various types of coping strategies. The child rated each item on a four-point scale. Example items included how often the child endorsed that they: “Draw, write or read something” and “Pray”. A mean score was used in the current study, with greater scores indicating more frequent use of the various types of coping strategies. In the current study sample, this measure demonstrated adequate internal consistency (i.e., α = 0.81).

###### Perceived Control of the Future.

This is a seven-item scale (Whitaker et al., 2000) that assessed a child's disposition-oriented beliefs about their control of the future, a construct closely tied to general self-efficacy (Bandura, 1977a). Items were rated on a four-point scale. Example items include, “I can do just about anything I set my mind to do” “What happens to me in the future mostly depends on me”, “It's really no use worrying about the future, because what will be will be”, and “My future is what I make of it”. A mean score was used in the current study, with higher scores indicating greater perceived control of the future. The internal consistency estimate (α) of this scale was 0.63.

##### Resilience Assets—Family Resources

The following scale was used to assess a possible resilience-related asset in the caregiving domain:

###### Trusting Relationship Questionnaire.

This scale (Mustillo et al., 2005) was comprised of 15-items that assessed the child's perceptions of whether they had a quality relationship with their current caregiver. Items were rated on a five-point scale. Example items include, “Do you share personal information about yourself with ‘caregiver'?” and “Do you enjoy spending time with ‘caregiver'?”. A mean score was used as a predictor in the current study, with higher scores indicating a higher quality, trusting relationship between the child and current caregiver. This measure demonstrated good internal consistency (α = 0.86).

##### Resilience Assets—Community Resources

The following scale was used to reflect broad community assets related to social support (e.g., peer, teacher):

###### Multi-dimensional Scale of Perceived Social Support.

This scale was adapted from the original scale created by Zimet et al. (1988) to add items assessing support from teachers and to reduce the number of Likert-style response options (i.e., five-point scale rather than seven-point scale). The full scale consisted of 16 items with four subscales reflecting social support from family, friends, teachers and other sources (i.e., a “special person”). Items were rated on a five-point scale. Example items include, “I can count on my friends when things go wrong”, “I can talk about my problems with my family” and “My teachers really try to help me”. In the current study, a mean score for each subscale (i.e., family support, friend support, teacher support, other support) was used as a predictor, with higher scores indicating increased levels of social support. These subscales demonstrated adequate internal consistency (α = 0.70 −0.75).

### Analytic Plan

Analysis was conducted using the SEARCH algorithm in STATA, a binary segmentation program for exploratory data analysis (Morgan and Sonquist, 1963; Sonquist et al., 1973; Morgan, 2005). As described in Sonquist et al. (1973, p. 11), “[SEARCH] divides the sample, through a series of binary splits into a mutually exclusive series of subgroups…They are chosen so that at each step in the procedure, the two new means account for more of the total sum of squares (reduce the predictive error more) than the means of any other pair of subgroups.” SEARCH has been coined an “automatic interaction detector” (Sonquist et al., 1973), given its utility in detecting complex, multiway interactions. While exploratory in nature, SEARCH also incorporates theory by selecting among researcher-specified, candidate predictors, here specified per the conceptual framework of risk and resilience for Chinese children affected by parental HIV, to optimally account for unexplained variance in child future orientation and future expectations.

One SEARCH run was conducted for each specified outcome, for a total of two runs of analysis. Each analysis was conducted with the same set of potential predictors described above. Program defaults were used to reduce the error variance in each outcome. For a split to occur, the minimum increase in explanatory power was set at 0.80%. The maximum number of splits and the fewest number of cases allowable in a subgroup were set at 25. Analyses were conducted on cases that had complete data across all selected predictors and the outcome.

## Results

### SEARCH Algorithm Results

**Figure 1:**
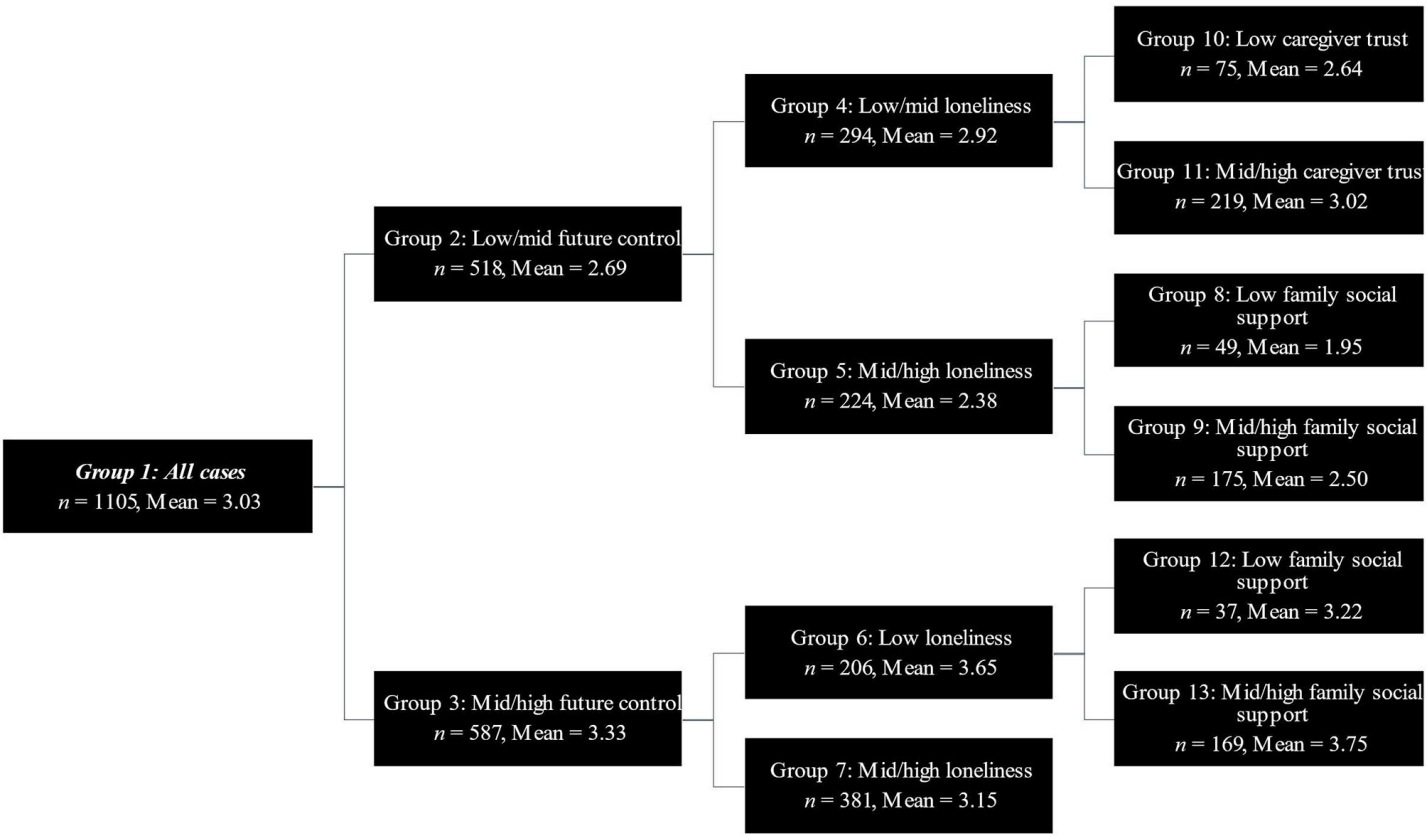
SEARCH-ing for future e**x**pectation. The decision tree for the outcome future expectation.

**Figure 2:**
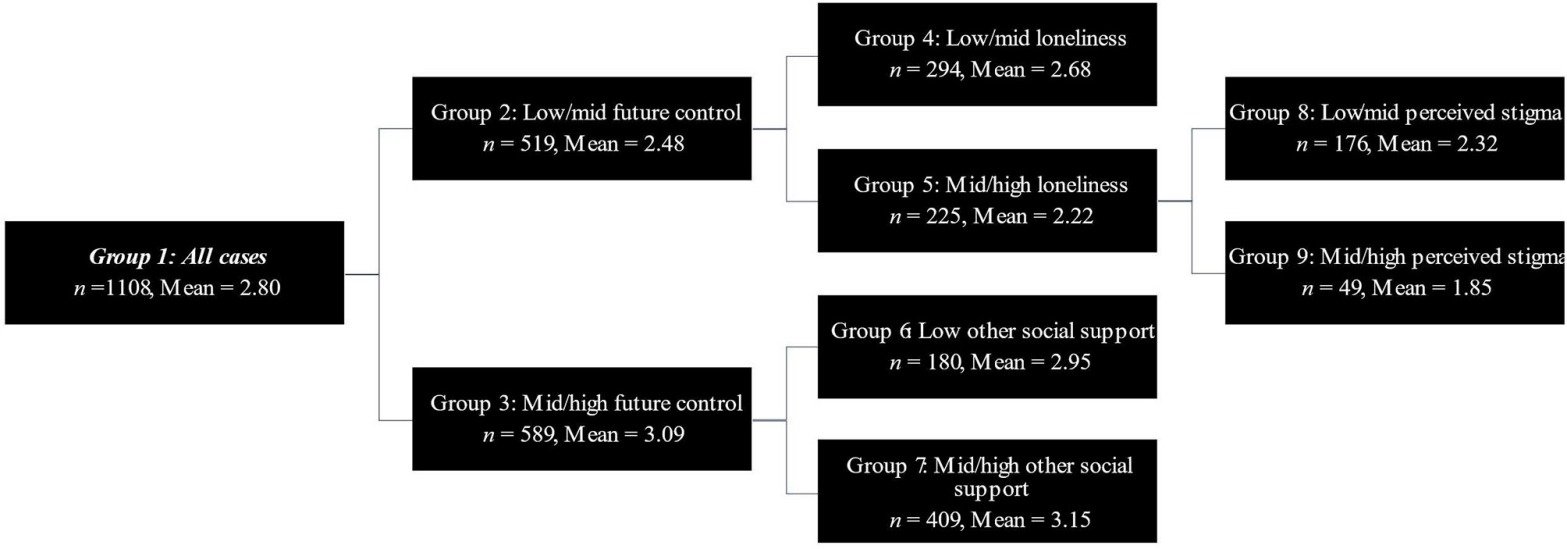
SEARCH-ing for future orientation. The decision tree for the outcome future orientation.

Demographic data for the full sample and descriptive statistics for all SEARCH predictors are shown in Tables 1, 2, respectively. Results from the SEARCH binary segmentation are presented in Tables 3, 4 and shown in Figures 1, 2. Key algorithm results for the two future-oriented outcomes are described below.

**Table 3:** SEARCH-ing for future expectation: final search groups, descriptive statistics, and mean differences.

**Final group**	**Description**	**N**	**Future expectation**		**Tukey HSD** ***post-hoc*** **test (Hedge's** ***g*** **[95% CI])**					
			***M***	***SD***	***v. Gr 8***	***v. Gr 9***	***v. Gr 10***	***v. Gr 11***	***v. Gr 12***	***v. Gr 13***
Gr 7	Mid-high future control; Mid-high loneliness	381	3.15	0.81	−1.21^**^ (−1.46) [−1.77 – −1.55]	−0.65^**^ (−0.77) [−0.96 to −0.59]	−0.51^**^ (−0.62) [−0.87 to −0.37]	−0.13 (−0.16) [−0.33 to 0.001]	0.06 (0.09) [−0.25 to 0.42]	0.59^**^ (0.78) [0.59 to 0.97]
Gr 8	Low-mid future control; Mid-high loneliness; Low family social support	49	1.95	0.89		0.55^**^ (0.61) [0.29 −0.93]	0.70^**^ (0.78) [0.40 – 1.15]	1.08^**^ (1.38) [1.05 – 1.71]	1.27^**^ (1.42) [0.94 to 1.90]	1.80^**^ (2.48) [2.09 to 2.88]
Gr 9	Low-mid future control; Mid-high loneliness; Mid-high family social support	175	2.50	0.90			0.14 (0.16) [−0.11 −0.43]	0.52^**^ (0.63) [.43 −0.84]	0.72^**^ (0.80) [.44 – 1.16]	1.25^**^ (1.57) [1.33 to 1.81]
Gr 10	Low-mid future control; Low-mid loneliness; Low caregiver trust	75	2.64	0.89				0.38^*^ (0.48) [0.23 to 0.75]	0.57^*^ (0.65) [0.25 to 1.05]	1.10^**^ (1.49) [1.19 to 1.79]
Gr 11	Low-mid future control; Low-mid loneliness; Mid-high caregiver trust	219	3.02	0.75					0.20 (0.26) [−0.09 to 0.61]	0.73^**^ (1.02) [0.81 to 1.23]
Gr 12	Mid-high future control; Low loneliness; Low family social support	37	3.22	0.90						0.53^*^ (0.74) [0.38 to 1.10]
Gr 13	Mid-high future control; Low loneliness; Mid-high family social support	169	3.75	0.67						

* *p < 0.01*,

** *p < 0.001*.

**Table 4:** SEARCH-ing for future orientation: final search groups, descriptive statistics, and mean differences.

**Final group**	**Description**	***N***	**Future orientation**		**Tukey HSD** ***post-hoc*** **test (Hedge's** ***g*** **[95% CI])**			
			***M***	***SD***	***v. Gr 6***	***v. Gr 7***	***v. Gr 8***	***v. Gr 9***
Gr 4	Low-mid future control; low-mid loneliness	294	2.68	0.69	0.26^**^ (0.42) [0.23 to 0.60]	0.47^**^ (0.77) [0.62 to 0.93]	−0.36^**^ (−0.49) [−0.68 to −0.30]	−0.84^**^ (−1.15) [−1.46 to −0.84]
Gr 6	Mid-high future control, low other social support	180	2.95	0.58		0.20^*^ (0.36) [0.19 −0.54]	−0.62^**^ (−0.90) [−1.12 to −0.69]	−1.10^**^ (−1.67) [−2.02 to −1.32]
Gr 7	Mid-high future control, high other social support	409	3.15	0.54			−0.82^**^ (−1.32) [−1.51 to −1.12]	−1.30^**^ (−2.22) [−2.54 to −1.89]
Gr 8	Low-mid future control, mid-high loneliness, low-mid perceived stigma	176	2.32	0.80				−0.48^**^ (−0.57) [−0.89 to −0.25]
Gr 9	Low-mid future control, mid-high loneliness, mid-high perceived stigma	49	1.85	0.89				

* *p < 0.01*,

** *p < 0.001*.

#### Future Expectation

The overall analytic sample mean (*n* = 1,105) for future expectation was 3.03 as rated on the five-point scale. The SEARCH algorithm was conducted with the full array of potential predictors described above. The run resulted in six binary splits on four predictors (i.e., future control, loneliness, caregiver trust, and family social support) and seven final groups (see Figure 1; final groups 7–13). Altogether, this model accounted for 22.78% of the variance in future expectations. A one-way ANOVA suggested a significant main effect of final group membership on future expectation, *F*(6, 1,098) = 53.97, *p* < 0.001. See Table 3 for final group descriptive statistics, between-group effect size estimates (i.e., Hedge's *g*) and *post hoc* comparisons using Tukey's Honestly Significant Difference (HSD) *post hoc* test. Notably, group 13, which consisted of children reporting mid-high control of the future, low loneliness, and mid-high family social support, reported the highest mean level of future expectation (*M* = 3.75, *SD* = 0.67, *N* = 169). Group 8, which consisted of children reporting low-mid future control, mid-high loneliness, and low family social report, reported the lowest levels of future expectation (*M* = 1.95, *SD* = 0.89, *N* = 49).

#### Future Orientation

The overall sample mean (*n* = 1,108) for future orientation was 2.80, as rated on a 4-point scale. The SEARCH algorithm was conducted with the full array of potential predictors described above. The run resulted in four binary splits on four predictors (i.e., future control, loneliness, other social support, and perceived stigma) and five final groups (see Figure 2; final groups 4, 6–9). Altogether, these binary splits accounted for 23.15% of the variance in future orientation. A one-way ANOVA suggested a significant main effect of final group membership on future orientation, *F*(4, 1,103) = 83.06, *p* < 0.001. See Table 4 for final group descriptive statistics, between-group effect size estimate (i.e., Hedge's *g*), and *post hoc* comparisons using Tukey's HSD *post hoc* test. Children in group 7, who reported mid-high control of the future and mid-high other social support, also demonstrated the highest mean levels of future orientation (*M* = 3.15, *SD* = 0.54, *N* = 409). Alternatively, children in group 9, who reported low-mid control of the future, mid-high loneliness, and mid-high perceived stigma, reported the lowest mean levels of future orientation (*M* = 1.85, *SD* = 0.89, *N* = 49).

## Discussion

While parental HIV places affected children at risk for negative social, emotional, behavioral, and educational outcomes (Li et al., 2008; Cluver et al., 2013), not all children demonstrate negative developmental trajectories (e.g., Chi et al., 2014a). Recent work has emphasized the need for a “shift from a deficit perspective to strength perspective” (Li et al., 2015, p. 218) to focus on resilience in children impacted by parental HIV. In particular, resilience-related assets at the child, family, and community levels have been identified as important for helping to buffer the risks associated with parental HIV, including HIV-related stigma (Li et al., 2015). The present research aimed to identify factors that interact to bolster positive future beliefs that have been identified as important resilience-related internal assets (Bandura, 1977a,b; Masten, 2011; Li et al., 2015).

Exploratory binary segmentation analyses identified several important predictors that interacted to predict children's future beliefs, including their perceptions about control of the future, trust in and support from others (e.g., caregivers, other family members, peers), feelings of loneliness, and perceptions of stigma. In the first exploratory analysis, children who reported high levels of perceived control of the future, low levels of loneliness, and high levels of family social support had the highest mean level of positive expectations about the future, such as expecting that they will have a happy life and be able to successfully cope with future challenges. Conversely, the group of children with the lowest mean levels of future expectation were children with low perceived control of the future, high levels of loneliness, and low family support. In the second analysis, children that had high perceived control over the future and reported high social support demonstrated the highest mean levels of future orientation. Alternatively, children that reported low perceived control of the future, high levels of loneliness, and high levels of perceived stigma against children impacted by parental HIV demonstrated the lowest mean levels of future orientation.

In line with current literature (Betancourt et al., 2013; Chi et al., 2015), this research suggests that many children impacted by parental HIV demonstrate important resilience-related assets. For instance, more than half of children in the current sample reported that they believed that it was “possible” or “very possible” that they would achieve specific positive outcomes in the future (e.g., graduate high school, achieve a good job, own a car, have a nice place to live). This suggests, that within this at-risk sample impacted by parental HIV, many children maintain a positive future outlook despite their challenging life circumstances (e.g., parental illness and separation, socio-economic disadvantage, community stigma).

Further, findings from our analyses suggest that, it may be important to jointly target several support systems external to the child, as well as internal resilience-related assets, to promote positive future-oriented beliefs for this vulnerable group of children. Child control of the future has been identified as an important factor that is promotive of positive child psychosocial outcomes in at-risk children in China (Zhang et al., 2009; Su et al., 2017). In the current study, children's perceptions about control over their future, a construct closely tied to self-efficacy, were associated with positive future beliefs. It is important to note here that conceptually and statistically (i.e., see bivariate correlations in Table 2), future expectation, orientation, and control are related but distinct constructs (i.e., all *r*s < 0.5). While the future expectation scale assesses a generalized belief about the future (e.g., “How sure are you that you will have a happy life?”) and the future orientation scale assesses specific expectations about socioeconomic attainment (e.g., “How likely do you think it is that you will graduate from high school or get your GED some day?”), the future control scale assesses how the child's efforts, capabilities, and agency are specifically linked to their future (e.g., My future is what I make of it”). This suggests a potentially important role for self-efficacy—or an individual's beliefs about their ability to perform a given task—in promoting resilience among this population (Bandura, 1977a; Betz and Hackett, 1981). At least one community-based intervention has been developed for children affected by parental HIV in South Africa that specifically targets children's self-efficacy; the Make A Difference (MAD) about Art program in South Africa aims to increase self-efficacy among children and adolescents (ages 8–18 years) affected by parental HIV, with children assigned to a quasi-experimental intervention condition showing increases in self-reported self-efficacy when compared to a comparison group that did not take part in the intervention (Mueller et al., 2011). This intensive intervention (i.e., 50+ sessions over a 6-month period) was led by trained and supervised peer ‘youth ambassadors' who delivered therapeutic art and psychoeducation content, with an emphasis on self-advocacy and empowerment (Mueller et al., 2011).

Moreover, children demonstrating greater perceived control of the future and greater social support (i.e., family or other social support from a “special person”), demonstrated the highest levels of future orientation. Findings in the current study highlighted important roles for loneliness and social support for children affected by parental HIV, with low levels of loneliness and increased family or other social support associated with more positive future beliefs. Previous research has shown that parental HIV infection can be associated with social maladjustment and disruptions in important social supports (for review see Chi and Li, 2013). For example, parental HIV can strain parenting behaviors and caregiver-child relationships (Rochat et al., 2017). The current study emphasizes the potential protective role of supportive familial and/ or caregiver-child relationships for children made vulnerable by HIV and shows a role for these factors in helping children to adopt future-oriented beliefs. Previous research has also underscored the importance of social support for children impacted by parental HIV (e.g., Hong et al., 2010). The present research extends this to highlight how different types of social support (e.g., family support, support of a “special person”) interact with other internal assets (e.g., perceived control of the future) to promote positive future orientation.

At the community-level, results suggest that perceived community stigma is detrimental to child wellbeing. This aligns with literature that indicates that perceived and experienced stigma are associated with poorer child outcomes, including poorer mental health (Chi et al., 2014b; Domlyn et al., 2020). However, it should be noted that only perceived stigma, and not experienced stigma, was selected for inclusion as a predictor in the SEARCH algorithm. These findings further suggest that perceived stigma is an important risk factor, with new evidence that perceived stigma interacts with other factors to reduce future orientation. Much of the intervention research to date has targeted a general population and focused on reducing stigmatizing beliefs surrounding infected HIV individuals (Brown et al., 2003). Less intervention research has focused on reducing stigma surrounding affected, but not infected family members (Brown et al., 2003; Chi et al., 2014b). While the present study was not an intervention study, it may inform potential targets for future prevention and intervention efforts. Future research should explore additional strategies to reduce community stigma impacting children affected by parental HIV, paying particular attention to the additional factors that may interact with stigma, notably loneliness and perceived control of the future.

Recently, a multi-level intervention designed to promote resilience among children affected by parental HIV has been developed based on Li and colleagues' conceptual framework of resilience (Li et al., 2015); the *Child-Caregiver-Advocacy Resilience* (*ChildCARE*) intervention aims to reduce risk and bolster positive outcomes among children affected by parental HIV in China by intervening at the child, caregiver, and community level. Recent evaluation of the intervention using a cluster-based randomized controlled trial and following enrolled children over a 3-year period has found the intervention to be efficacious in improving a number of key psychosocial outcomes for both children and caregivers (Harrison et al., 2017, 2018, 2019; Li et al., 2017). Findings from the current study could potentially be used to optimize targeting of internal assets and social support in this and other interventions for Chinese children affected by HIV. For instance, future research could explore the utility of screening children in areas identified by the current algorithm (e.g., social support, feelings of loneliness, perceived stigma) prior to delivering the intervention so that the program could be tailored for different risk groups.

### Limitations and Future Directions

There are several limitations to the current research that are important to consider. The future-oriented outcomes were examined in a cross-sectional manner and the research was not conducted in an experimental or quasi-experimental manner to account for potential confounders. As such, these analyses are not meant to be interpreted in a causal manner. Future work could lend additional insight into the relationships amongst these constructs by examining the relationships over time. Additionally, the estimated internal consistency for a few of the candidate predictors fell slightly below recommended cutoffs (i.e., Cronbach's alpha ≥ 0.7). This may be due to our use of brief measures for some variables, as alpha depends both upon magnitude of the correlations of items as well as the number of items in the scale (Cronbach, 1951). Future work should explore interactions among the same constructs with measures demonstrating more ideal psychometric characteristics. Moreover, we did not explore how measurement properties may differ across development. Although the outcome variables and many of the predictor variables demonstrated adequate reliability in the sample as a whole, measurement properties may have varied by demographic characteristics. Additional measurement work in this vulnerable sample seems warranted.

Further, while this study leverages a large cohort of children affected by HIV, the SEARCH analyses may have been improved by use of a larger sample size, as exploratory data techniques benefit from very large sample sizes (i.e., 1,000 or more; Sonquist et al., 1973). Future work could explore these relationships in larger samples by combining samples from several studies conducted across various contexts utilizing an integrative data analysis (Hussong et al., 2013). An integrative data analysis, using innovative methods such as moderated non-linear factor analysis (Bauer, 2017), may also overcome some of the aforementioned measurement shortcomings related to measurement in the current study (Hussong et al., 2020). Similarly, it will be important to consider additional candidate predictors in future research. For example, research on parental loss suggests that loss of a mother, father, either, or both may be associated with differential outcomes (Berg et al., 2016). In the current study, we did not explore the possible differential impacts of the death of a mother vs. father but this and other variables will be important to explore in future research.

Additionally, these analyses were exploratory in nature to understand how resilience-related assets interacted to promote future orientation. As additional datasets are collected on this population, cross validation will be warranted to further support the robustness of relationships identified in the current research. In addition, complementary exploratory analytic strategies merit exploration in this substantive area, such as considering latent constructs with the use of SEM Trees (Brandmaier et al., 2016). Finally, the analyses here were conducted only in a sample of youth affected by parental HIV in rural China. As such, it is unknown if the results generalize to youth impacted to parental HIV in other contexts or to youth unaffected by parental HIV. Additional work should consider broader samples to ascertain the limits of generalizability.

## Conclusions

The current research, through interaction detection, explicated several important factors that work together to promote future-oriented beliefs among youth impacted by parental HIV in China. Results indicated that children's perceptions about their control of the future, trust in and support from others (e.g., caregivers, other family members, peers), feelings of loneliness, and perceptions of stigma interact to impact future-oriented beliefs, an important, resilience-related belief. Knowledge of how these factors operate in tandem may inform future applied research focused on appropriate screening and tailored prevention and intervention programming to ultimately optimize outcomes for youth impacted by parental HIV in China.

## Data Availability Statement

The datasets presented in this article are not readily available because of the sensitive nature of the data and to ensure participant privacy. Requests to access the datasets should be directed to XL, Professor and SmartState Endowed Chair for Clinical Translational Research, XIAOMING@mailbox.sc.edu.

## Ethics Statement

The studies involving human participants were reviewed and approved by Wayne State University in the United States and Beijing Normal University in China. Written informed consent to participate in this study was provided by the participants' legal guardian/next of kin.

## Author Contributions

HM, SH, AF, and XL contributed to conception and design of the study. HM organized the database, performed the statistical analysis, and wrote the first draft of the manuscript. SH wrote sections of the manuscript. All authors contributed to manuscript revision, read, and approved the submitted version.

## Funding

This work has been supported, in part, by funding from the James N. Morgan Fund for New Directions in Analysis of Complex Interactions, Institute of Social Research, University of Michigan, as well as by funding from the National Institutes of Health under award number: R01MH076488.

## Author Disclaimer

The opinions expressed are those of the authors and do not represent views of the funders.

## Conflict of Interest

The authors declare that the research was conducted in the absence of any commercial or financial relationships that could be construed as a potential conflict of interest.

## Publisher's Note

All claims expressed in this article are solely those of the authors and do not necessarily represent those of their affiliated organizations, or those of the publisher, the editors and the reviewers. Any product that may be evaluated in this article, or claim that may be made by its manufacturer, is not guaranteed or endorsed by the publisher.
